# Supplementary material for: Amine-Functionalized Natural Halloysite Nanotubes Supported Metallic (Pd, Au, Ag) Nanoparticles and Their Catalytic Performance for Dehydrogenation of Formic Acid
Source: Nanomaterials (Basel). 2022 Jul 14;12(14):2414. doi: 10.3390/nano12142414 (PMC9318759; doi:10.3390/nano12142414)
Supplement: Supplementary file 1 [file nanomaterials-12-02414-s001.zip › nanomaterials-1798870-supplementary.pdf]

nanomaterials

MDPI

Supporting Information

Amine-Functionalized Natural Halloysite Nanotubes Supported Metallic (Pd, Au, Ag) Nanoparticles and Their Catalytic Performance for Dehydrogenation of Formic Acid

Limin Song [1], Kaiyuan Tan [1], Yingyue Ye [1], Baolin Zhu [1,2,*], Shoumin Zhang [1,2] and Weiping Huang [1,2,*]

1 College of Chemistry, Nankai University, Tianjin 300071, China; 17853135525@163.com (L.S.); tky8246159357@163. com (K.T.); aniy1110@hotmail.com (Y.Y); zhangsm@nankai.edu.cn (S.Z.)
2 The Key Laboratory of Advanced Energy Materials Chemistry (Ministry of Education), Nankai University, Tianjin 300071, China
* Correspondence: zhubaolin@nankai.edu.cn (B.Z.); hwp914@nankai.edu.cn (W.H.)

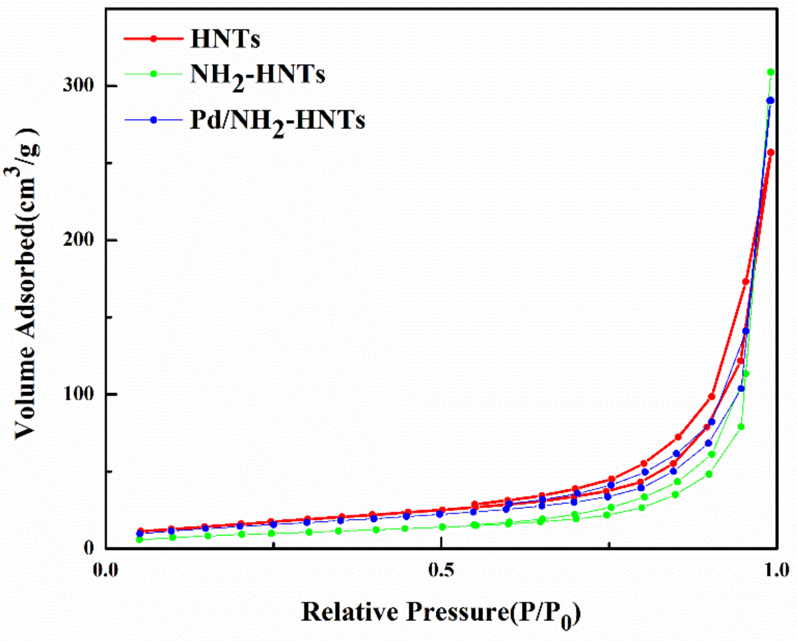

Figure S1. N_2 adsorption desorption isotherms of various samples.

Table S1. Positions and assignments of the IR vibration bands

Position (cm^{-1})	Assignments
3701	O-H stretching of inner-surface hydroxyl groups
3626	O-H stretching of inner hydroxyl groups
2930	symmetric stretching of $C\text{-}H_2$
1631	O-H deformation of water
1570	deformation (scissoring) of $N\text{-}H_2$
1490	deformation (scissoring) of $C\text{-}H_2$
1330	deformation (scissoring) of Si-CH
1105	perpendicular Si-O stretching
910	O-H deformation of inner hydroxyl groups

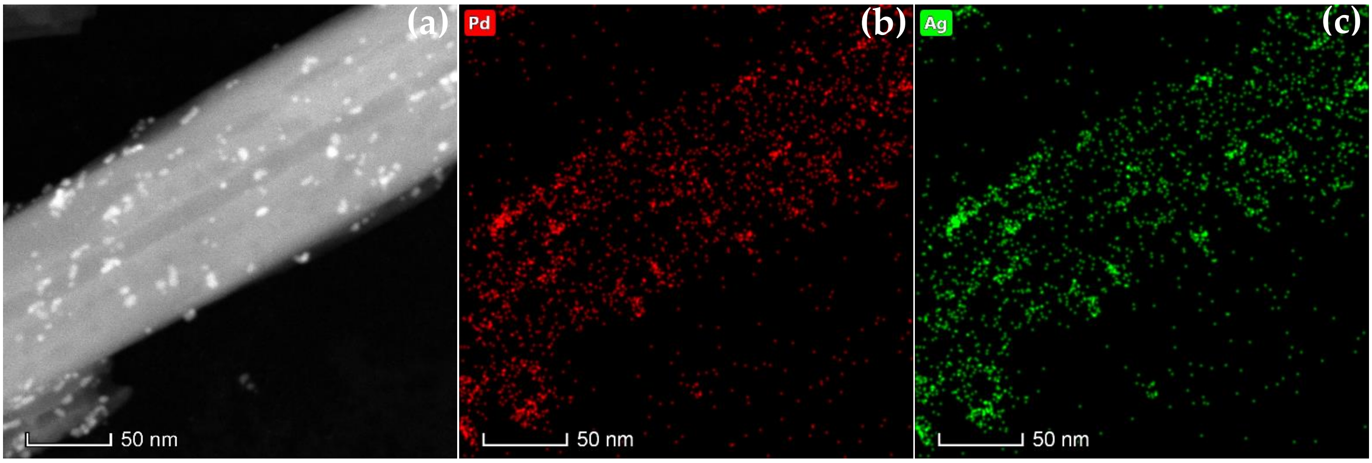

Figure S2. **(a)** HAADF-STEM images of PdAg-NH2-HNTs; **(b)** EDX mapping of Pd element in PdAg/NH2-HNTs; **(c)** EDX mapping of Ag element in PdAg/NH2-HNTs.

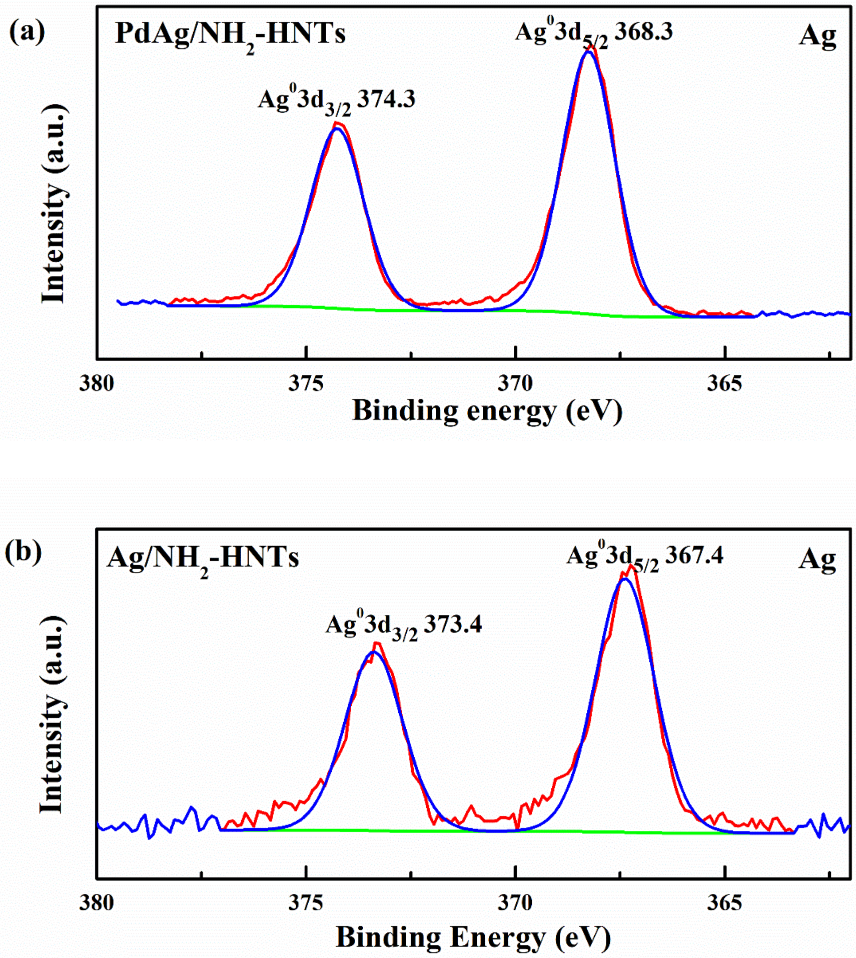

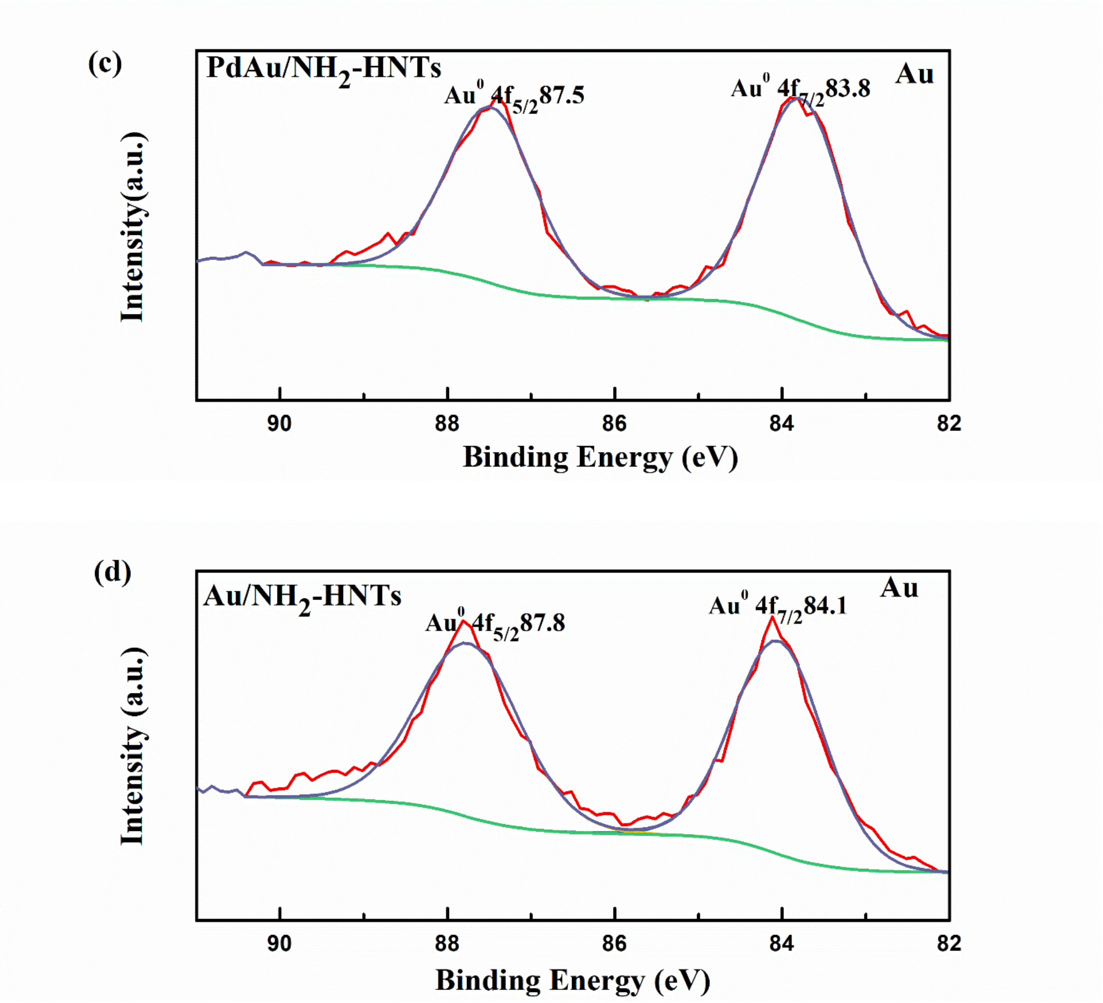

Figure S3. Ag 3d XPS spectra of **(a)** PdAg/NH2-HNTs; **(b)** Ag/NH2-HNTs; Au 4f XPS spectra of **(c)** PdAu/NH2-HNTs; **(d)** Au/NH2-HNTs.

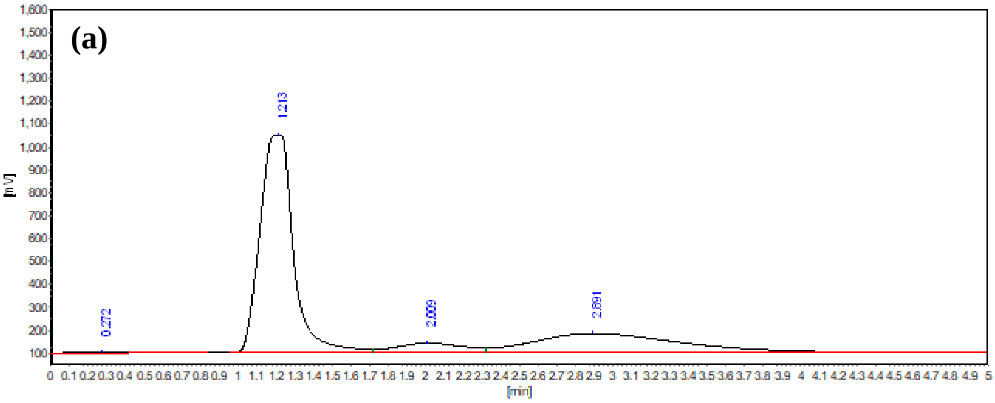

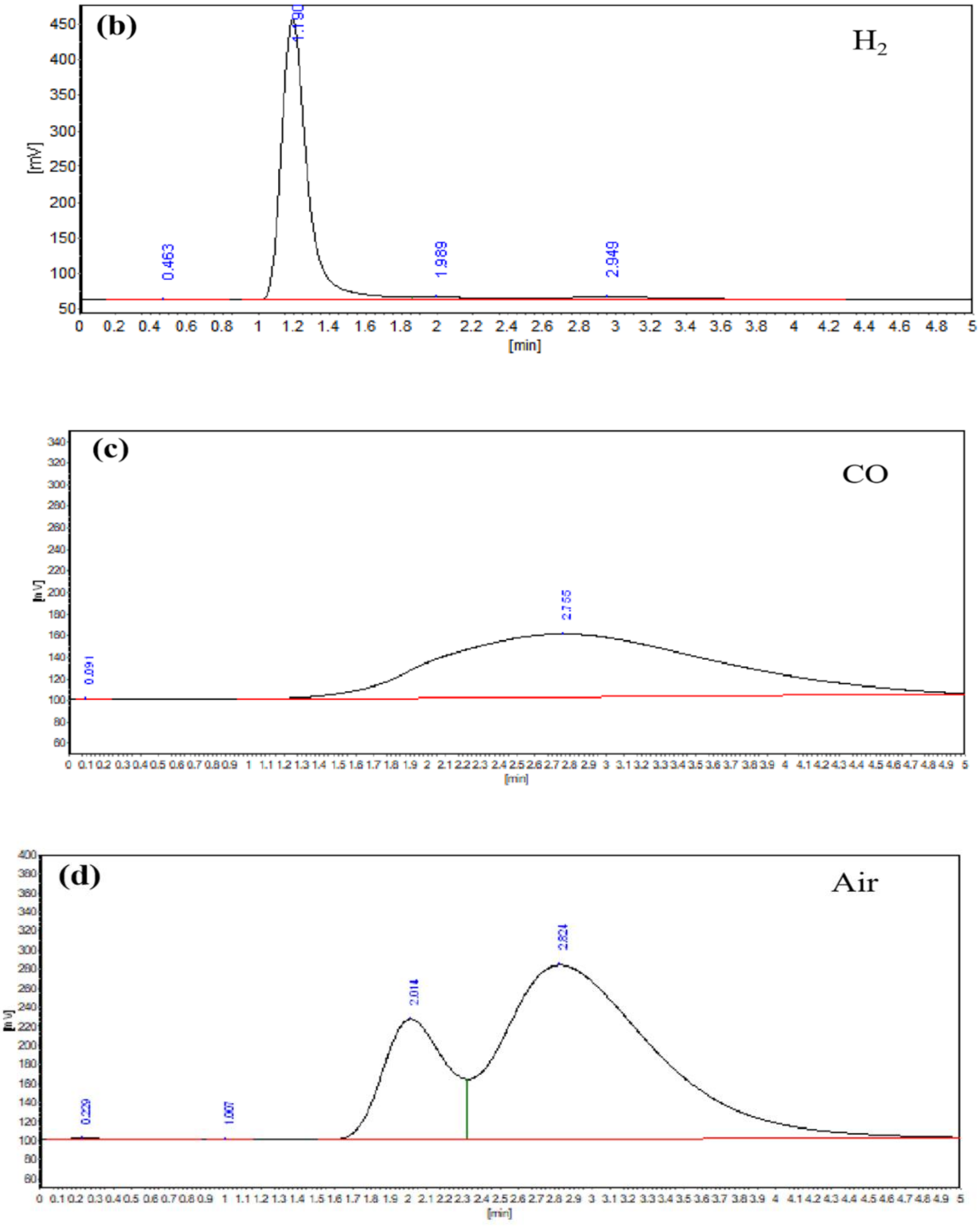

Figure S4. GC results of (**a**) Pd/NH_2-HNTs and standard sample (**b**) H_2, (**c**) CO and (**d**) air.

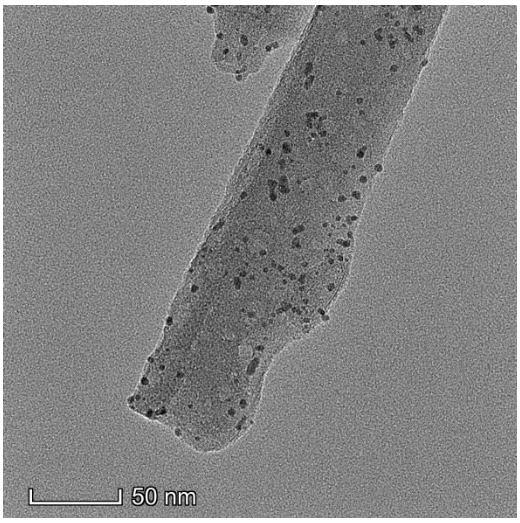

Figure S5. The TEM images of spent Pd/NH_2-HNTs catalysts after fifth catalytic reaction cycle.

Table S2. Turnover frequency (TOF) for different catalysts used for the generation of dihydrogen.

Catalysts	Metal Loading/%	Metal Size (nm)	T/K	TOF/h^{-1}	Ref
Pd/NH_2-HNTs	Pd (2.5)	1.8±0.4	298	412.9	This work
Pd/S-1-in-K	Pd (0.64)	1.5	298	856	[1]
Au1Pd1.5/MIL-101-NH_2	Au (14.18) Pd (11.49)	2.2 ± 0.5	298	526	[2]
Pd/mpg-C3N4	Pd (9.5)	1.7	298	144	[3]
Pd/NH_2-KIE-11	Pd (5.9)	1.6	298	860.7	[4]
Pd/NMC-400	Pd (3.2)	2.7 ± 0.6	298	913	[5]

References

1. Wang, N.; Sun, Q.; Bai, R.; Li, X.; Guo, G.; Yu, J. In situ confinement of ultrasmall Pd clusters within nanosized silicalite-1 zeolite for highly efficient catalysis of hydrogen generation. *J. Am. Chem. Soc.* **2016**, *138*, 7484.
2. Cheng, J.; Gu, X.; Liu, P.; Zhang, H.; Ma, L.; Su, H. Achieving efficient room-temperature catalytic H_2 evolution from formic acid through atomically controlling the chemical environment of bimetallic nanoparticles immobilized by isoreticular amine-functionalized metal-organic frameworks. *Appl. Catal. B: Environ.* **2017**, *45*, 1953–1958.
3. Lee, J.H.; Ryu, J.; Kim, J.Y.; Nam, S.W.; Han, J.H.; Lim, T.H.; Gautam, S.; Chae, K.H.; Yoon, C.W. Carbon dioxide mediated, reversible chemical hydrogen storage using a Pd nanocatalyst supported on mesoporous graphitic carbon nitride. *J. Mater. Chem. A* **2014**, *2*, 9490–9495.
4. Lee, D.W.; Jin, M.H.; Park, J.H.; Lee, Y.J.; Choi, Y.C.; Chan Park, J.; Chun, D.H. Alcohol and water free synthesis of mesoporous silica using deep eutectic solvent as a template and solvent and its application as a catalyst support for formic acid dehydrogenation. *ACS Sustain. Chem. Eng.* **2018**, *6*, 12241–12250.
5. Sun, J.; Qiu, H.; Cao, W.; Fu, H.; Wan, H.; Xu, Z.; Zheng, S. Ultrafine Pd particles embedded in nitrogen-enriched mesoporous carbon for efficient H_2 production from formic acid decomposition. *ACS Sustain. Chem. Eng.* **2019**, *7*, 1963–1972.
